# Breakthrough electroneutron multi-response miniature dosimetry/spectrometry in medical accelerator

**DOI:** 10.1038/s41598-024-59871-1

**Published:** 2024-04-25

**Authors:** Mehdi Sohrabi, Maryam Malekitakbolagh, Hasan Ali Nedaei

**Affiliations:** 1 Health Physics and Dosimetry Research Laboratory, Department of Energy Engineering and Physics, Amirkabir University of Technology, Tehran, Iran; 2 Department of Radiotherapy Oncology, Cancer Institute, University of Medical Sciences, Tehran, Iran

**Keywords:** Electroneutron dosimetry/spectrometry, Miniature neutron dosimeter/spectrometer, Energy-specific/tissue-specific dosimetry, Spectrometry, High-energy electron medical accelerator, Physics, Applied physics

## Abstract

Breakthrough multi-response miniature dosimetry/spectrometry of electroneutrons (EN) was made on surface and in-depths of whole-body polyethylene phantom under 10 cm × 10 cm electron beam of 20 MV Varian Clinac 2100C electron medical accelerator commonly applied for prostate treatment. While dosimetry/spectrometry of photoneutrons (PN) has been well characterized for decades, those of ENs lagged behind due to very low EN reaction cross section and lack of sensitive neutron dosimeters/spectrometers meeting neutron dosimetry requirements. Recently, Sohrabi “miniature neutron dosimeter/spectrometer” and “Stripe polycarbonate dosimeter” have broken this barrier and determined seven EN ambient dose equivalent (ENDE) (µSv.Gy^–1^) responses from electron beam and from albedo ENs including beam thermal (21 ± 2.63), albedo thermal (43 ± 3.70), total thermal (64 ± 6.33), total epithermal (32 ± 3.90), total fast (112.00), total thermal + epithermal (l96 ± 10), and total thermal + epithermal + fast (208 ± 10.23) ENs. Having seven ENDE responses of this study and seven PNDE responses of previous study with the same accelerator obtained at identical conditions by the same principle author provided the opportunity to compare the two sets of responses. The PNDE (µSv.Gy^–1^) responses have comparatively higher values and 22.60 times at isocenter which provide for the first time breakthrough ENDE responses not yet reported in any studies before worldwide.

## Introduction

High energy medical linear accelerators, commonly operate in both electron or X-ray modes, have found worldwide applications for patient cancer radiotherapy. Electrons or X-rays of a prescribed dose will be delivered to a cancerous patient tumor site with well-planned treatment protocols. Electron or X-ray beams also produce unwanted neutrons through electroneutron (EN) or photoneutron (PN) reactions. The cross-section for PN production is approximately 137 times higher than that of the EN production^1,2^.

For over half a century, determination of PN ambient dose equivalents (PNDE) and EN ambient dose equivalents (ENDE) to patients and medical staff have been of high interest. The patients undergoing high energy electron or X-ray cancer therapy, receive unwanted ENDE or PNDE exposures which may cause second primary cancer (PN- or EN-SPC) risks. Therefore, determination of accurate thermal, epithermal and fast PNDE and/or ENDE of such unwanted neutrons is of high prime importance.

Accurate neutron dosimetry/spectrometry in mixed radiation fields in particular in high-energy/high-dose electron or X-ray beams of medical accelerators should meet certain neutron dosimetry requirements. Some requirements include high sensitivity to thermal, epithermal, and fast ENs or PNs emitted directly from the beam, albedo neutrons scattered back from the phantom and surrounding environment; insensitivity to high-dose high-energy of low-LET radiation (X, γ, β, electrons) and non-ionizing radiation; capability to separate thermal, epithermal and fast neutrons from either directions; high spacial resolution for surface and depth dose studies; capability to determine tissue-specific and energy-specific ENDE; little/negligible post-exposure fading; and easy to use large number of dosimeters on surface and in phantom depths under only one single exposure^3–5^. Consideration of such requirements are of crucial importance in particular for ENDE determination having very low cross sections.

PN dosimetry/spectrometry in medical accelerators has been advanced for over decades by us^3–10^, and by others^11–14^. However, due to having very low EN cross section; unavailability of neutron dosimeter/spectrometers meeting the requirements; and difficulty in obtaining electron beam free time have made EN dosimetry/spectrometry rather limited.

Albeit the limitations, some studies have used detectors such as pure gold activation and TLD(600/700) in Bonner spheres^1,2^; bubble/CR-39 detectors^15–20^; nested neutron spectrometer^21^ and simulation by MCNP^16,18^. However, most of such studies due to some limitations can only report total ambient dose equivalents mostly at the isocenter, without separating thermal, epithermal and fast ENs or PNs from the beam and from albedo neutrons scattered back to the dosimeter. Accordingly, it can be concluded for the first time in this study that “No matter how accurate each ENDE or PNDE determination has been performed (usually at the isocenter), neutron dosimeters applied by others mostly report only one value of total neutron dose emitted from the beam plus albedo neutrons within its energy sensitivity range”. Therefore, the results of such studies even if accurately determined are over-estimates of values emitted from the beam. So the ENDE or PNDE results commonly reported should be carefully considered when used for cross section calculations, PN- EN -SPC risk estimation, etc.

In order to address the many deficiencies in such measurements, we believe that the “Sohrabi miniature neutron dosimeter/spectrometer’ (hereafter miniature dosimeter/spectrometer) and “Stripe polycarbonate neutron dosimeter (Stripe PND or PND) can fulfill the requirements as successfully applied in our studies^4,5,22^, as well described in the method’s section. This miniature neutron dosimeter/spectrometer can successfully determine seven EN energy-specific and tissue-specific ambient neutron dose equivalent (ENDE) responses including thermal, epithermal and fast neutrons directly emitted from the beam as well as albedo thermal, epithermal and fast neutrons scattered back from the phantom and surrounding environments, and also total thermal, total epithermal, total fast, total thermal + epithermal and total thermal + epithermal + fast ENs. This miniature neutron dosimeter/spectrometer is rather simple and small with high spacial resolution for tissue-specific and energy-specific dosimetry with nil sensitivity to high-energy high-dose electrons and X-rays (see experiments and methods). The spacial resolution of the method is high enough such that large number of dosimeters can be placed on surface and in phantom depth and even around the accelerator head, all to be exposed under one single exposure. Since the miniature dosimeter/spectrometer has nil fading, they can be also processed and evaluated in time. Therefore, the dosimeter is a good candidate for ENDE and PNDE studies. Having said the above, it is the purpose of this paper to:

1. Determine seven energy-specific and tissue-specific ENDE ($\mu$ Sv.Gy^–1^) responses of 20 MeV electron dose (Gy) on surface and in depths of a polyethylene (PE) human-size phantom, including beam thermal, albedo thermal, total thermal, total epithermal, total fast, total thermal + epithermal and total thermal + epithermal + fast EN responses in the electron beam of 20 MeV electron beam of a Varian Clinac 2100C,
2. Apply and demonstrate the “Sohrabi miniature neutron dosimeter/spectrometer” and “Stripe PND” for determining ambient dose equivalent components of extremely low level electroneutron doses in such exotic applications, and
3. Compare and discuss the seven ENDE ($\mu$ Sv.Gy^–1^) responses of this study with the seven PNDE responses of 18 MV X-ray beams of the same accelerator performed under identical conditions, as published by the principle author in this journal^4^.

### Experiments and methods

**Figure 1:**
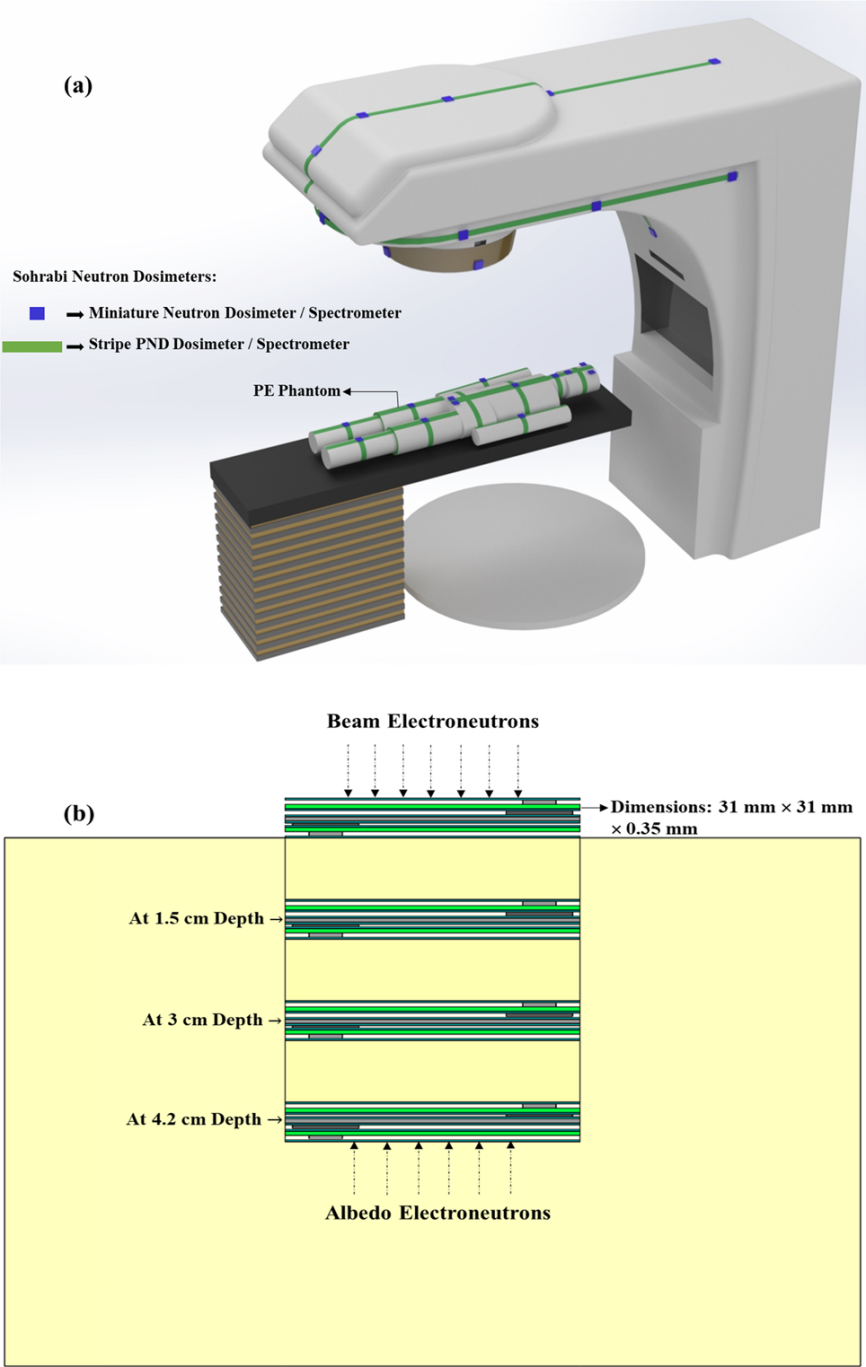
(**a**) Schematic diagram of Varian Clinac 2100C medical linear accelerator with PE phantom on the patient couch with two types of miniature dosimeter/spectrometer and Stripe PND arranged on the surface of the phantom on different organs for a 10 cm × 10 cm field for prostate cancer with gonads at the isocenter, and (**b**) the pelvis partial PE phantom with 4 miniature dosimeters/spectrometers at 0.0, 1.5, 3.0 and 4.2 cm depths.

The seven ENDE responses were determined in 10 cm × 10 cm electron beam of a 20 MV Varian Clinac 2100C medical linear accelerator; the same accelerator also used by us recently for obtaining detailed PNDE responses^4^. All the experiments were performed on a human-sized polyethylene (PE) phantom on which two types of Sohrabi neutron dosimeters were placed. Also at the center of the pelvis partial phantom, a cylindrical hole has been carved so that miniature dosimeter/spectrometers can be installed in depth with PE cylindrical spacers between them. Figure 1a,b shows; (a) schematic view of the medical linear accelerator with PE phantom on the patient couch under the beam on which two types of dosimeters have been placed, and (b) pelvis partial PE phantom with 4 miniature dosimeters/spectrometers placed at 0.0, 1.5, 3.0 and 4.2 cm depths.

For ENDE determination, “miniature neutron dosimeter/spectrometers” and “Stripe PNDs” were used. Details on these dosimeters have been given in our publications in particular the recent ones^4,5,7^. However, a summary of such developments follow:

The “PNDs” have been in use since the discovery of enlarging fast-neutron-induced particle tracks in polycarbonate by ECE processing to a point observed by the unaided eyes^23^. The basic dosimetry characteristics of the PNDs such as adequate dose range, energy response matching ICRP ambient dose equivalent energy response, angular dependence, low background tracks, no fading, application as stripes in worker’s belt, etc.^24^. In particular, by inventing multi-detector ECE chamber^25^, large-scale applications such as neutron individual dosimetry, radon monitoring indoors and outdoors as well as other applications have been advanced, in particular for pioneering PN studies in 3 medical accelerators at the Emory University in USA^6,7^ and in Radiotherapy Department of Omeed Hospital in Isfahan^26^. The PNDs can be used in different sizes as desired cut from large sheets available in different thicknesses such as 250, 500, and 1000 µm from polymer markets. The favorable characteristics invited many applications requiring different PND sizes such as 3 cm × 3 cm as used also in this study, 2 to 3 m long stripes for radon indoor monitoring on the walls^27^, mega-size radon dosimetry by a mega-size ECE chamber^27,28^, “PN volume dose equivalent” hypothesis and methodology by mega-size PNDs^29^, etc.

The PND, however, has disadvantage of no sensitivity to neutrons with energy < 1 MeV. By inventing an albedo neutron dosimeter with ^6^Li/PND in cadmium covers through ^6^Li(n_th_,α)^3^H reaction (cross section; 946 barn), made the dosimeter also highly sensitive to albedo thermal, epithermal and fast neutrons^22^. The PND is also highly sensitive to alpha particles in particular 1.47 MeV thermal/epithermal-neutron-induced alpha particles from ^10^B converter through ^10^B(n_th_,α)^7^Li reaction (3840 barn) as used in the miniature dosimeter/spectrometer^30,32^.

**Figure 2:**
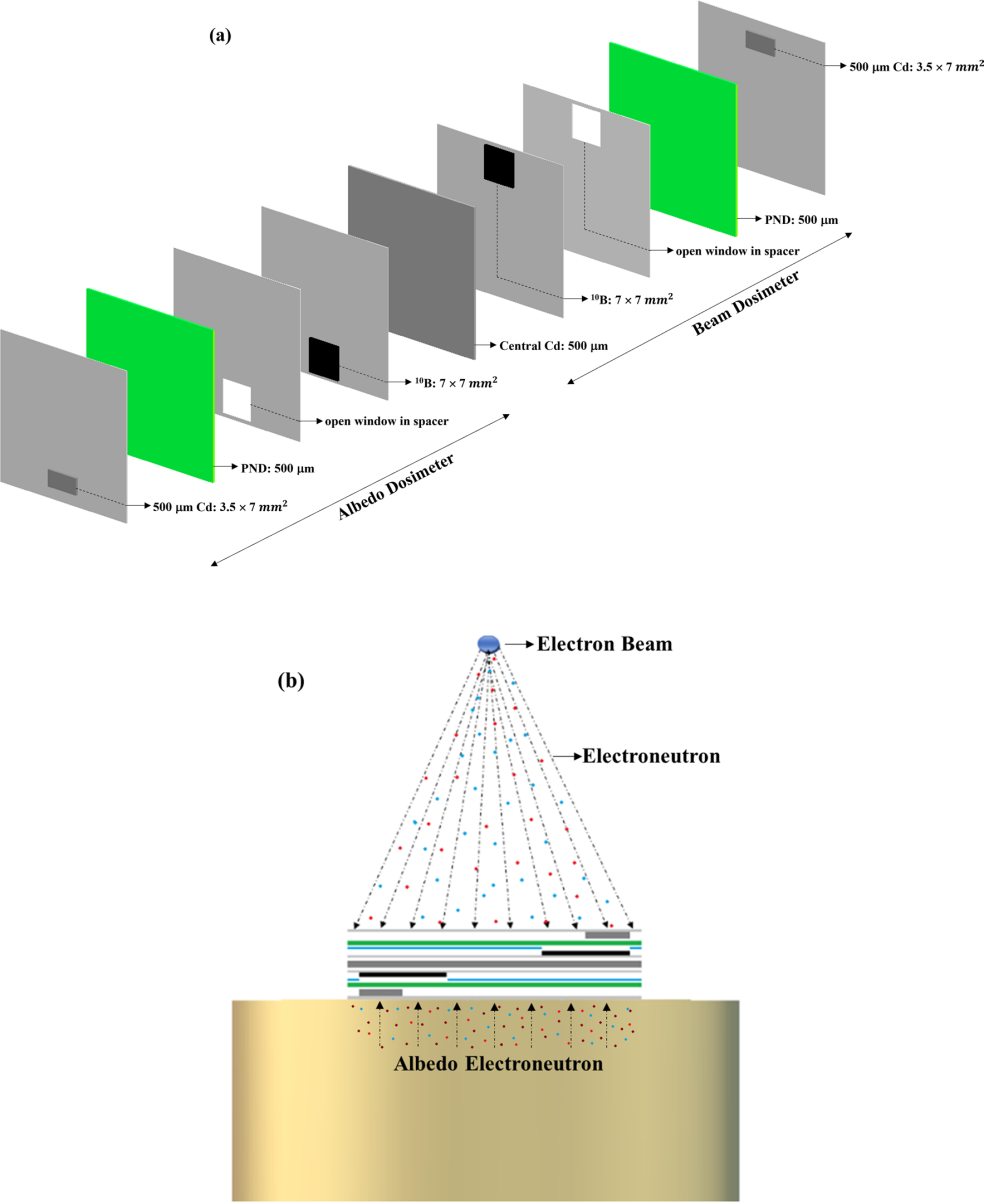
(**a**) Schematic components of the “miniature neutron dosimeter/spectrometer”, and (**b**) the dosimeter components assembled in a badge as placed on a phantom.

The miniature dosimeter/spectrometer has been well detailed in our recent articles^4,5^. Figure 2a,b shows; (a) schematically the components of the miniature dosimeter/spectrometer, and (b) the dosimeter components assembled in a badge as placed on a phantom.

Briefly speaking, as shown in Fig. 2a, the dosimeter consists of two similar albedo dosimeters separated by a cadmium foil with structure as follow:

1. One central 0.5 mm cadmium foil which basically separates two albedo dosimeters from each other and also shields thermal neutrons entering from either side passing to the other dosimeter; one dosimeter facing the beam and the other facing the phantom,
2. Each albedo dosimeter on either side has 500 µm thick PND (one side of it faces the central cadmium and the other side faces 0.7 cm × 0.7 cm size enriched ^10^B foil; half of which faces a cadmium chip 3.5 mm × 7.0 mm to allow stopping thermal neutrons and only passing epithermal neutrons to be detected. This PND in each dosimeter therefore detects fast neutrons from either side, epithermal neutrons from either side and thermal neutrons separately from either side; from the direct neutron beam and from albedo neutrons.
3. After neutron exposure, the PNDs were processed by ECE to amplify the fast-neutron-induced recoil and alpha particle tracks. The tracks of each section of the PND can be precisely counted by well experienced eyes. Then the track density of each section was converted to ambient dose equivalent by using the relevant conversion factors, as well described before^4,5^.

The “miniature dosimeter/spectrometer”, as described fulfils the requirements for neutron dosimetry in such exotic electron or 18 MV X-ray beams. In particular, the dosimeter can well determine seven ENDE responses of beam thermal, albedo thermal, total thermal, total epithermal, total fast, total thermal + epithermal and total thermal + epithermal + fast ENs. Therefore, they were successfully applied in obtaining seven ENDE responses and also to obtain seven PNDE responses^4,5^ in high-dose high-energy electron or X-ray beams.

Details on the calibration of the dosimeters have been well covered in our previous studies^24,30^. However, briefly speaking, for determining energy response of the PND, a number of precisely calibrated neutron sources (e.g. fission neutron spectrum; Pu-Be; 16, 35 and 50 MeV d + on Be targets from 3 different cyclotrons) have been used demonstrating that PND neutron energy response match well with ICRP ambient dose equivalent H*(10) from ∼1 to ∼20 MeV^24,30^. A constant conversion factor was obtained over the stated energy range making the PND response independent of neutron spectrum. In order to calibrate the dosimeter with albedo design, it was also calibrated by a standard ^252^Cf source. For thermal and epithermal PN dose equivalent determination, conversion factors were also obtained from extensive fast, epithermal and thermal neutron dosimetry studies of the albedo neutron dosimeters using PND/^10^B (with or without cadmium cover). The dosimeter was calibrated on different phantoms with different calibrated neutron sources such as Pu-Be, Am-Be and in particular ^252^Cf source, in a scatter free air environment, as well as by simulation^30,32^.

## Experimental results

Seven major energy-specific and tissue-specific ENDE (µSv) per Gy electron dose responses were determined at some major locations on the surface and in depths of PE pelvis phantom. The electron dose was delivered at the isocenter to gonad’s site and in pelvis depths at the source-to-skin distance (SSD) of 100 cm under 10 cm × 10 cm field of a Varian Clinac 2100C electron medical linear accelerator. The seven ENDE (µSv.Gy^–1^) responses for beam thermal, albedo thermal, total thermal, total epithermal, total fast, sum total thermal + epithermal and sum total thermal + epithermal + fast ENs were determined. In a recent study published by us in this journal^4,5^, seven energy-specific and tissue-specific PNDE (µSv.Gy^–1^) responses of 18 MV X-rays of the same accelerator at the same conditions have been determined. Having available seven PNDE (µSv.Gy^–1^) in 18 MV X-ray beam of the same accelerator beam, the two sets of seven ENDE and seven PNDE responses were also compared, since there is no such data available for comparison in the literature. Therefore, this section includes two subsections on “Electroneutron whole-body ambient dose equivalent distributions on surface and in phantom depths” and **“**Comparison of the seven ENDE and PNDE responses”, as follow.

### Electroneutron whole-body surface and in-depth ambient dose equivalent distributions

**Figure 3:**
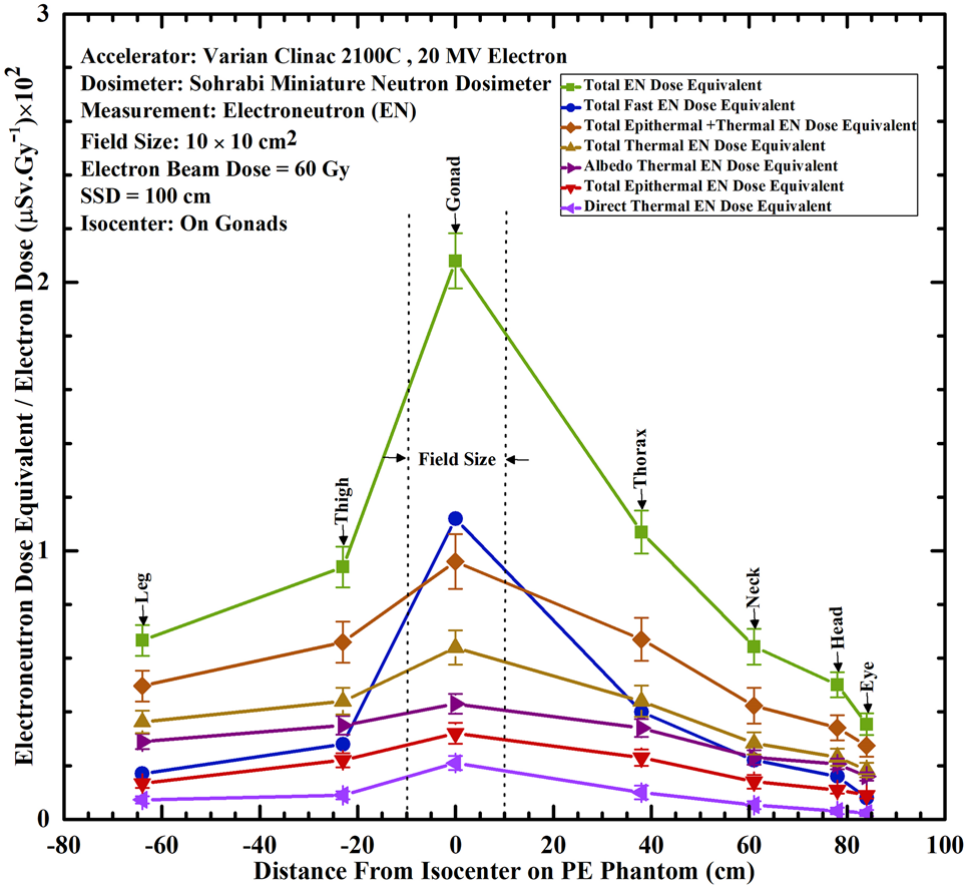
Seven ENDE/Gy 20 MeV electron dose (µSv.Gy^–1^) versus distance from isocenter (gonads) responses on different locations on surface for beam thermal, albedo thermal, total thermal, total epithermal, total fast, total thermal + epithermal and total thermal + epithermal + fast ENDEs.

Seven ENDE (µSv.Gy^–1^) of 20 MeV electron dose versus distance responses on gonad’s site at the isocenter obtained by the miniature dosimeter/spectrometer placed on phantom surface for beam thermal, albedo thermal, total thermal, total epithermal, total fast, total thermal + epithermal and total thermal + epithermal + fast ENDEs. The electron dose was delivered in two separate 30 Gy exposures to obtain total electron dose of 60 Gy of 20 MeV electrons at a the SSD of 100 cm. The high 60 Gy electron dose was delivered since the ENDE values at some locations far from the isocenter on the phantom were expected to be very low. The data obtained for the eyes, gonads, thighs and legs are mean values of two similar measurement locations on the phantom organs. The seven ENDE (µSv)/Gy 20 MeV electron dose responses versus distance from isocenter on phantom surface are shown in Fig. 3.

As shown in Fig. 3, the miniature dosimeter/spectrometer demonstrates how well ENDE multi-responses can be resolved and differentiated from each other so that at any location, relevant ENDE values can be extracted. It also shows how well the ENDE responses in terms of energy on the PE phantom surface can be analyzed.

It can be seen that the values of fast ENDE (µSv.Gy^–1^) response dominate the values of other responses in and outside the beam; it is the major ENDE component of the total ENDE response. Therefore, the fast ENDE response follows the same trend as the total ENDE response. On the other hand, the beam thermal ENDE response is smaller than that of the albedo thermal ENDE response but they follow the same trend outside the beam. The percentage of the other ENDE values relative to total ENDE value at the isocenter was analyzed to be; beam thermal (10.1%), albedo thermal (20.67%), total thermal (30.76%), total epithermal (15.86%), total fast (53.37%), and total thermal + epithermal (46.63%), all of which can be physically and conceptually explained. Such examples show how well the miniature dosimeter/spectrometer determines the seven ENDE components and how well differentiates the single ENDE values and responses in and out of the beam of such medical accelerators.

In our recent multi-directional PN spectrometry studies made using our 6 PND/^10^B detectors on a cube placed in each of PE spheres published in this journal^10,31^, the full PN energy spectrum at the isocenter of 18 MV X-ray beam and in some other locations outside the beam in a radiotherapy bunker characterized two peaks; one thermal PN peak with an epithermal and intermediate energy tail followed by another peak for fast PNs around ~ 1 MeV. In another EN spectrometry study^21^, using a nested neutron spectrometer, EN spectrum also demonstrates a similar PN spectrum trend as obtained for PNs by us^10,31^; i.e. “one thermal PN peak with an epithermal and intermediate energy tail followed by another peak for fast PNs around ~ 1 MeV (of course with a lower fluence than that of PN). On the other hand, as seen in Fig. 3, the responses obtained in this EN study also shows a total thermal EN of 30.76%, and a total fast EN of 53.37% almost consisting with the major peaks of the PN and EN spectra. Therefore, the dosimeter/spectrometer by this simple miniature spectrometry is in confirmation with the PN and EN spectra discussed above^10,21,31^.

**Figure 4:**
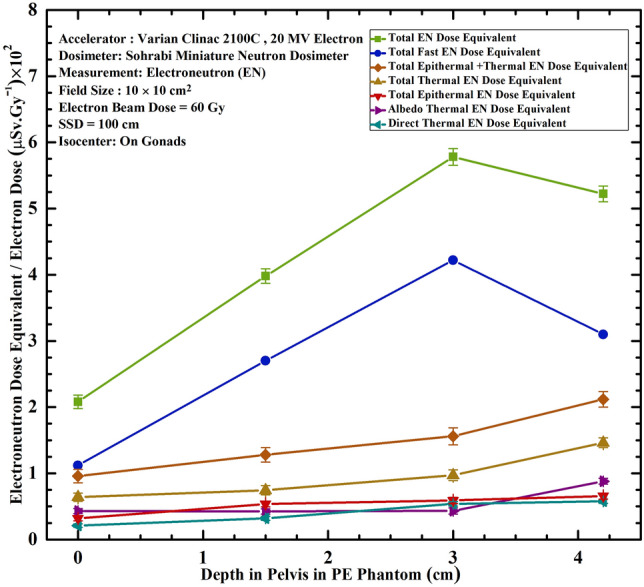
Seven ENDE per Gy 20 MeV electron dose (µSv.Gy^–1^) versus depth responses in PE pelvis phantom for thermal, albedo thermal, total thermal, total epithermal, total fast, total thermal + epithermal and total thermal + epithermal + fast ENDEs under 10 cm × 10 cm electron beam on gonads.

In-depth ENDE (µSv.Gy^–1^) response studies require a neutron dosimeter with high spacial resolution and sensitive to detect all seven ENDE components so that it can be easily used in locations in depth where space is limited. For this purpose, a cylindrical hole has been carved at the center of pelvis PE phantom such that the dosimeters could be embedded at different tissue depths with PE cylindrical spacers. The miniature dosimeter/spectrometer with 4 cm × 4 cm × 0.3 dimensions has among many unique features very high spacial resolution meeting the requirements for this purpose. It can also determine seven ENDE in-depth energy-specific and tissue-specific responses uniquely, not yet explored by other dosimeters so far. Therefore, the miniature dosimeter/spectrometer also meets the necessary requirements as applied at different depths of the PE pelvis phantom placed at the isocenter of an electron beam. Figure 4 shows seven in-depth ENDE (µSv.Gy^–1^) 20 MV electron dose versus distance responses at 4 pelvis depths of the PE phantom at the isocenter (gonads) for beam thermal, albedo thermal, total thermal, total epithermal, total fast, total thermal + epithermal and total thermal + epithermal + fast ENDEs.

Figure 4 well demonstrates the behavior of ENs at different depths of the PE pelvis phantom showing seven ENDE energy-specific tissue-specific values for each depth. The fast ENDE contribution of the total ENDE response in depth is the major ENDE to tissue peaking at 3 cm depth. Since the fast ENDE response dominates in the beam, the total ENDE response follows the same trend as fast EN response. On the other hand, the thermal and epithermal ENDE responses increase in value as depth in phantom increases since fast ENs are thermalized by passing though the depth of the phantom. Thermal ENDE response of the beam is smaller than that of the ENDE albedo thermal response but they follow the same trend. Such examples show that how the miniature dosimeter/spectrometer differentiates the seven ENDE energy-specific and tissue-equivalent responses inside and outside the beam as well as in-depth of the PE phantom.

**Figure 5:**
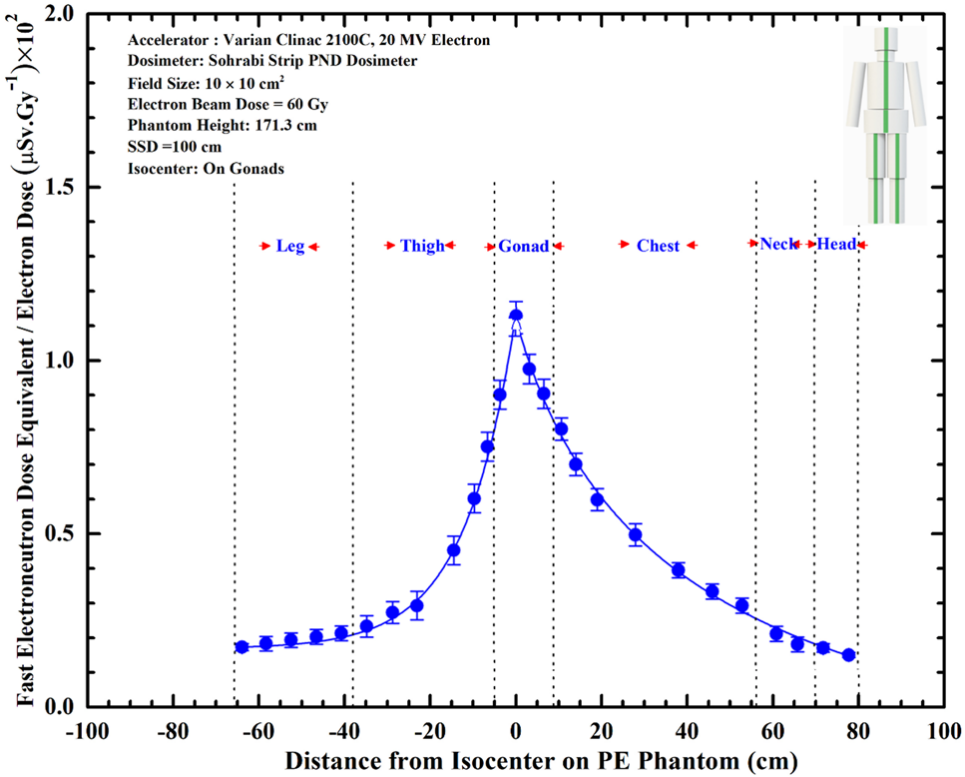
Fast ENDE (µSv.Gy^–1^) per Gy electron dose versus the distance from the isocenter on the surface of the PE phantom.

In order to determine also fast ENDE response on the phantom surface and around an organ, Stripe PNDs were used. The PND in general is a unique dosimeter which can be used in any sizes from small to mega sizes and most commonly as Stripe dosimeters^4,5,24,27^. It can be easily laid over and/or wrapped around each organ. Figure 5 shows ENDE (µSv.Gy^–1^) per 20 MeV electron dose versus distance from isocenter on the surface of the PE phantom.

**Figure 6:**
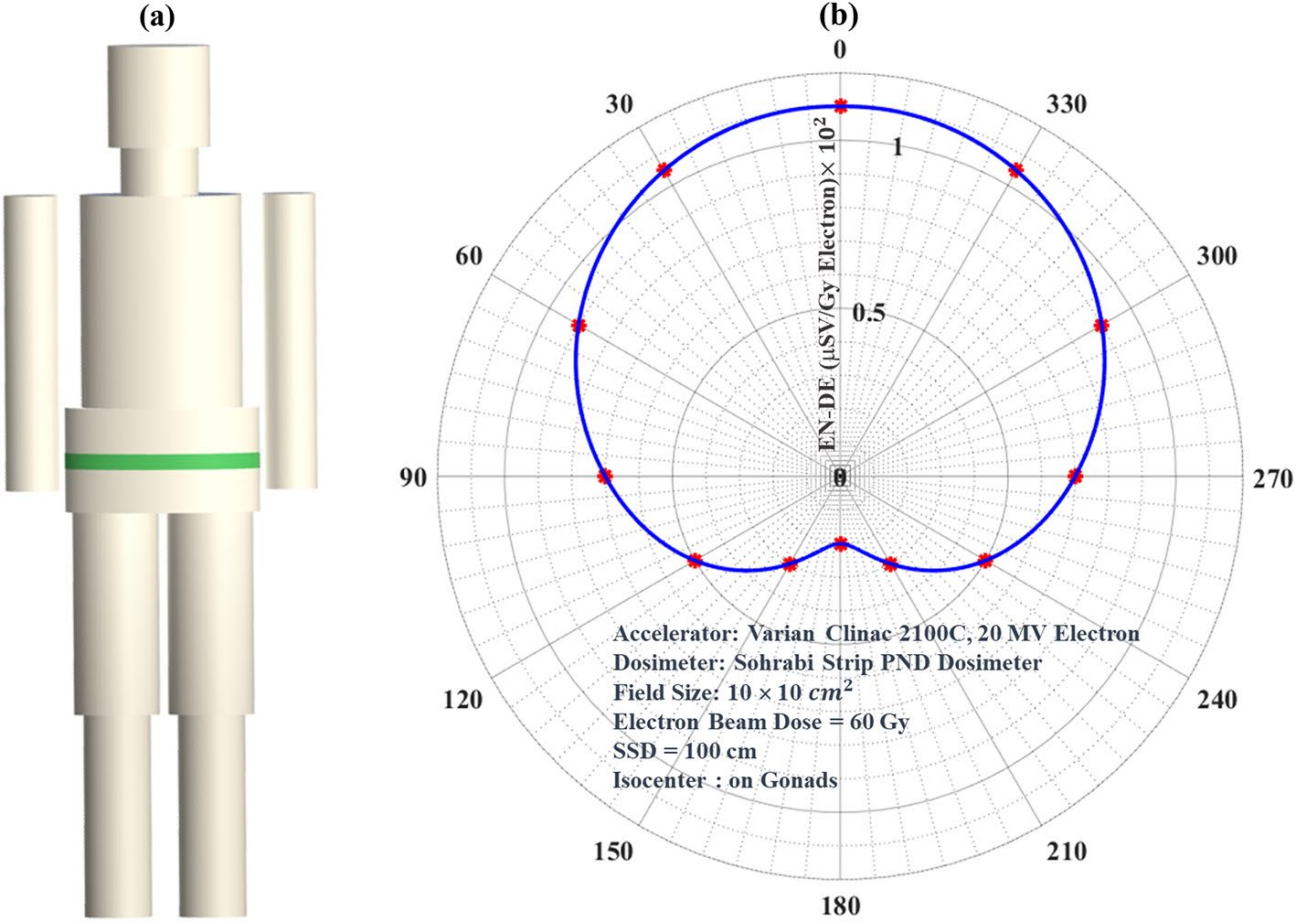
(**a**) The PE phantom with wrapped Stripe PNDs around organs in particular pelvis and (**b**) 360° angular surface ENDE distribution showing the orientation dependence of the surface ENDE values.

The Stripe PNDs have many superior characteristics as stated above in particular flexibility to easily bend for any application requiring exotic geometric conditions such as wrapping the stripe around organs such as pelvis, legs, arms, head, etc. This characteristic has been well studied in our recent PNDE 360° angular dose equivalent distribution studies^5^. In this study, the Stripe PND with 3.1 cm width was wrapped 360° around the PE pelvis phantom. The angular fast ENDE distribution response (µSv.Gy^–1^) versus surface points around 360° angular distribution are shown in Fig. 6a,b. As can be seen, the PE pelvis with gonads being directly at the isocenter in the field receives maximum angular dose equivalent ENDE at 0° and minimum ENDE at 180^o^ between which values of different angles.

### Comparison of ENDE and PNDE ambient dose equivalent responses

**Figure 7:**
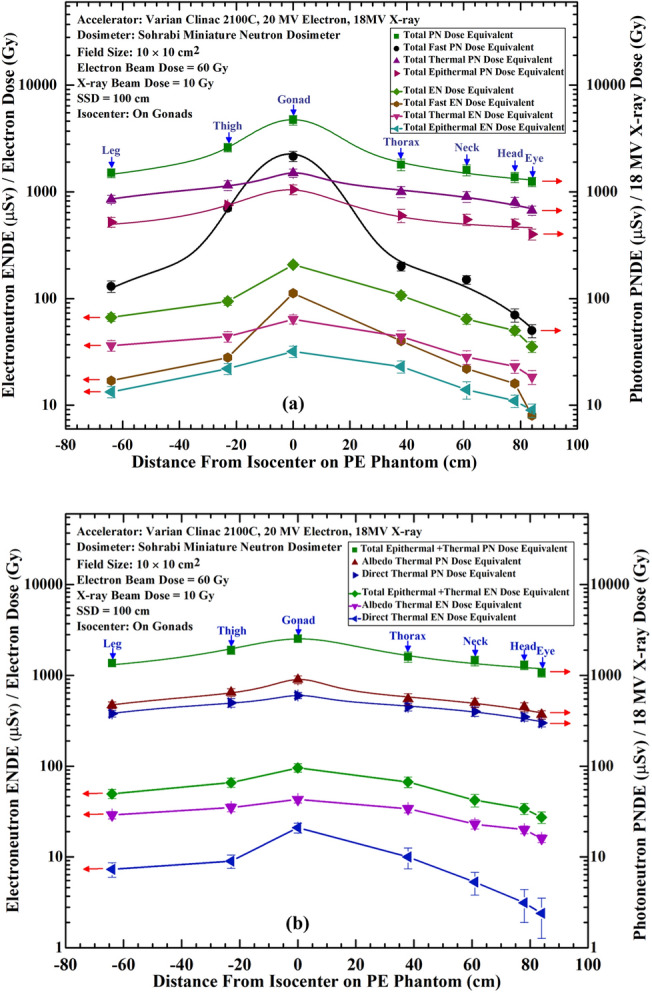
Comparison of seven ENDE (µSv.Gy^–1^) (left scale) with seven PNDE (µSv.Gy^–1^) (right scale) versus distance responses from isocenter for beam thermal, albedo thermal, total thermal, total epithermal, total fast, total thermal + epithermal and total thermal + epithermal + fast ENs and PNs. Figure (**a**) compares responses of beam thermal, albedo thermal, and total thermal + epithermal and Figure (**b**) compares responses of total thermal, total epithermal, total fast and total thermal + epithermal + fast ENs (µSv.Gy^–1^) with those of PNs (µSv.Gy^–1^)^4^.

As stated above, the seven ENDE (µSv.Gy^–1^) responses on the PE phantom surface for beam thermal, albedo thermal, total thermal, total epithermal, total fast, total thermal + epithermal and total thermal + epithermal + fast ENs were shown in Fig. 3. These seven unique ENDE (µSv.Gy^–1^) responses have been obtained for the first time in this study. On the other hand, as stated above, we have recently published in this journal seven PNDE (µSv.Gy^–1^) versus distance responses from isocenter in 18 MV X-rays beams using exactly the same exposure conditions^4^. Having such unique seven ENDE and seven PNDE responses, they were compared to demonstrate the relative values, as shown in Fig. 7a,b.

From Fig. 7a,b, it can be concluded that values of seven PNDE (µSv.Gy^–1^) per Gray 18 MV X-ray responses are much larger than those of ENDE (µSv.Gy^–1^) per Gray of 20 MeV electrons. For ease of comparison, some peak values of seven PNDE responses relative to seven ENDE responses are 19.20 times for total fast, 28.60 times for beam thermal, 20.90 times for albedo thermal, 23.43 times for total thermal, 26.56 times for total epithermal, 26.56 times for total thermal + epithermal, and 22.60 times for total thermal + epithermal + fast ENs. It can be well observed that the ratios of PNDE to ENDE responses at the isocenter are about 20 to 33 times larger, since the PN cross section is much larger than that of ENs^1,2^. This comparison is unique since all the conditions of the two sets of responses are completely identical. Such ratios can also be extracted for the seven responses on other points on the responses in Fig. 7a,b.

In order to facilitate easy comparison of the ENDE and PNDE response values at different locations, the data on Fig. 7a and b are given also in Table 1.

**Table 1:** Compares the ENDE and PNDE values of seven ENDE (µSv.Gy^–1^) with seven PNDE (µSv.Gy^–1^) versus distance responses from isocenter (gonads) for beam thermal, albedo thermal, total thermal, total epithermal, total fast, total thermal + epithermal and total thermal + epithermal + fast ENs and PNs^4^.

Organs	Comparison of 7 electroneutron dose equivalent (ENDE)* responses (This research) and photoneutron dose equivalent (PNDE)** responses at a Varian Clinac 2100C (60 Gy 20 MV Electrons & 10 Gy 18 MV Photoneutron) for a 10 cm × 10 cm at 100 cm at the isocenter on Gonads^4^													
	Leg		Thigh		Gonad (Isocenter)		Thorax		Neck		Head		Eye	
EN-DE & PN-DE per electron or X-ray dose	EN* (μSv/Gy)	PN** (μSv/Gy)	EN* (μSv/Gy)	PN** (μSv/Gy)	EN* (μSv/Gy)	PN** (μSv/Gy)	EN* (μSv/Gy)	PN** (μSv/Gy)	EN* (μSv/Gy)	PN** (μSv/Gy)	EN* (μSv/Gy)	PN** (μSv/Gy)	EN* (μSv/Gy)	PN (μSv/Gy)
Total fast neutron dose equivalent	17 ± 00	130 ± 16	28 ± 00	700 ± 39	112 ± 00	2150 ± 110	40 ± 00	200 ± 19	22 ± 00	150 ± 14	16 ± 00	70 ± 10	8 ± 00	50 ± 7
Direct thermal neutron dose equivalent	7 ± 1.3	380 ± 33	9.00 ± 1.50	500 ± 55	21.00 ± 2.63	600 ± 49	10.00 ± 2.58	450 ± 44	5.30 ± 1.48	400 ± 47	3.14 ± 1.24	350 ± 37	2.40 ± 1.13	300 ± 29
Albedo thermal neutron dose equivalent	29 ± 2.8	470 ± 41	35 ± 3.5	650 ± 64	43 ± 3.70	900 ± 65	34 ± 3.30	550 ± 77	23 ± 2.7	500 ± 57	20 ± 2	450 ± 49	16 ± 1.60	370 ± 38
Total epithermal neutron dose equivalent	13.36 ± 1.60	520 ± 55	22 ± 2.6	750 ± 80	32 ± 3.90	1050 ± 95	23 ± 3	600 ± 85	14 ± 2.60	550 ± 68	11 ± 1.46	500 ± 55	9 ± 1.30	400 ± 47
Total thermal neutron dose equivalent	36.30 ± 4.13	850 ± 74	44 ± 5	1150 ± 119	64 ± 6.33	1500 ± 114	44 ± 5.88	1000 ± 121	28 ± 4.18	900 ± 104	23.14 ± 3.24	800 ± 86	18.40 ± 2.73	670 ± 67
Total epithermal + thermal neutron dose equivalent	49.66 ± 5.73	1370 ± 129	66 ± 7.60	1900 ± 199	96 ± 10.23	2550 ± 209	67 ± 8.88	1600 ± 206	42.30 ± 6.78	1450 ± 172	34.14 ± 4.70	1300 ± 141	27.40 ± 4.03	1070 ± 114
Total neutron dose equivalent	66.66 ± 5.73	1500 ± 145	94 ± 7.60	2600 ± 238	208 ± 10.23	4700 ± 319	107 ± 8.88	1800 ± 225	64.30 ± 6.78	1600 ± 186	50.14 ± 4.70	1370 ± 151	35.40 ± 4.03	1120 ± 121

**Figure 8:**
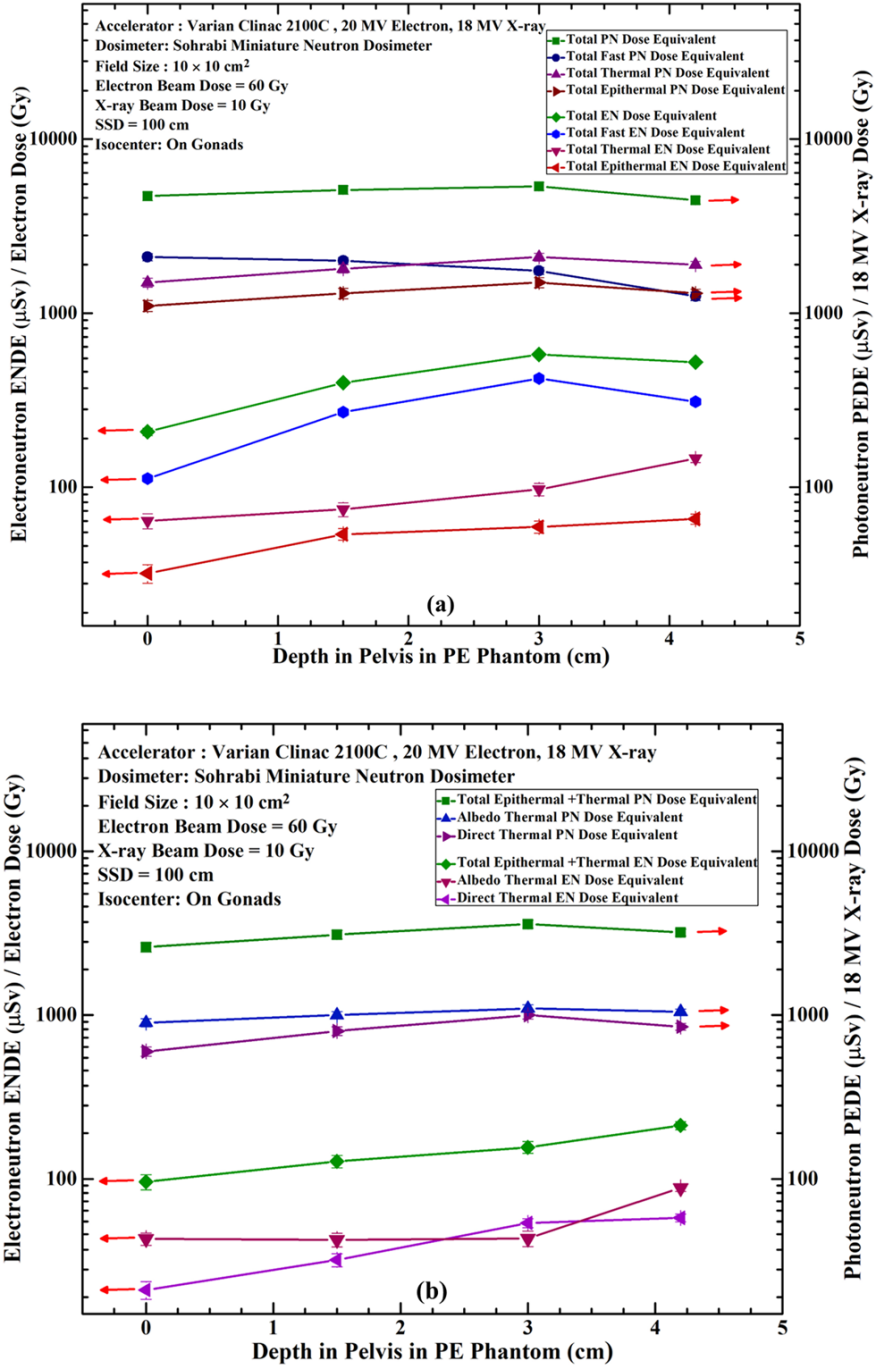
Comparison of seven ENDE (µSv.Gy^–1^) per Gray 20 MeV electron dose (left scale) with seven PNDE (µSv.Gy^–1^) 18 MV X-ray dose (right scale) at depths 1.0, 1.5, 3.0 and 4.2 cm in PE pelvis phantom at the isocenter; for total thermal, total epithermal, total fast and total thermal + epithermal + fast neutrons (**a**) and for beam thermal, albedo thermal and total thermal + epithermal neutrons (**b**)^4^.

Also Fig. 8a,b compares seven ENDE (µSv.Gy^–1^) per Gray 20 MeV electron dose with seven PNDE (µSv.Gy^–1^) per Gray 18 MV X-ray dose versus 0.0, 1.5, 3.0 and 4.2 cm depths in the PE pelvis for dosimeters located at the isocenter (gonads) for beam thermal, albedo thermal, total thermal, total epithermal, total fast, total thermal + epithermal and total thermal + epithermal + fast ENs (left scale) and PNs (right scale). Like Fig. 7a,b, Fig. 8a compares responses of total thermal, total epithermal, total fast and total thermal + epithermal + fast neutrons and Fig. 8b compares those of beam thermal, albedo thermal and total thermal + epithermal neutrons.

As also stated above, in order to facilitate easy comparison of the ENDE and PNDE response values at different locations, the data on Fig. 8a,b are provided in Table 2.

**Table 2:** Ratio of PNDE to ENDE at a Varian Clinac 2100C (60 Gy 20 MeV electron) & (10 Gy 18 MV x-rays for a 10 cm × 10 cm at SSD 100 cm on gonad’s locattion^4^.

Ratio of photoneutron dose equivalent to electroneutron dose equivalent at a varian clinac 2100C (60 Gy 20 MeV Electrons) & (10 Gy 18 MV X-rays) for a 10 cm × 10 cm at 100 cm on Gonads^4^						
Leg	Thigh	Gonad	Thorax	Neck	Head	Eye
7.65	25.00	19.20	5.00	6.81	4.37	6.25
52.05	55.56	28.60	45.00	75.47	111.46	125.00
16.20	18.57	20.93	16.18	21.74	22.50	23.12
38.92	34.10	32.81	26.09	39.28	45.45	44.44
23.42	26.14	23.43	22.73	31.80	34.57	36.41
27.59	28.79	26.56	23.88	34.28	38.08	39.05
22.50	27.66	22.60	16.82	24.88	27.32	31.64

Since the spectra of ENs^21^ and PNs^31^ have the same trends; i.e. one thermal PN or EN peak with an epithermal and intermediate energy tail followed by another peak for fast PNs or fast ENs around ~ 1 MeV. Therefore, they are expected to follow the same thermalizing process so that the seven ENDE and seven PNDE energy-specific and tissue-specific responses; i.e. beam thermal, albedo thermal, total thermal, total epithermal, total fast, total thermal + epithermal and total thermal + epithermal + fast ENs at depths tend to peak at 3 cm depth. The depth of ENDE or PNDE responses well proves the unique spacial characteristic of the dosimeter as well as potential to provide seven energy-specific and tissue-specific responses at each depth point. This is in fact a breakthrough ENDE response studies of miniature dosimetry/spectrometry even at different tissue depths; what has not been reported in other studies so far.

## Discussions

Determination of unwanted EN and PN ambient dose equivalent generated in the accelerator beams is of prime importance and interest to obtain data for protection of patients undergoing electron or X-ray therapy and radiation workers as well as for scientific reasons which has been in progress for decades. Measurement of such unwanted neutrons has been of interest to scientists, medical physicists, health physicists, physicists, regulatory authorities, etc. This is in particular very important since such ENs and PNs may cause PN- or EN-SPC risks to patients.

As stated in the introduction, the cross-section for PN generation is approximately 137 times higher than that for ENs^1,2^. Therefore, while determination of PNDE (µSv.Gy^–1^) has been advanced in large number of studies by us and others worldwide, those of ENDE (µSv.Gy^–1^) have been limited to few studies, in particular in our studies which provides seven energy-specific responses.

For PNDE or ENDE dosimetry in such exotic beams, the neutron dosimeter should have certain characteristics as given in the introduction. In particular, the dosimeter should be capable to determine thermal, epithermal and fast neutrons emitted directly from the beam as well as those scattered back into the dosimeter from albedo neutrons. The dosimeter should also be highly insensitive to high-energy high-dose X-ray or electron beams. Separation of ENDE responses at different neutron energy ranges delivered to patients is also of primary importance since the radiation weighting factor (W_R_) for neutrons highly depends on neutron energy^33^. For surface and in-depth dosimetry, having a neutron dosimeter with high spacial resolution is also a main requirement.

For ENDE dosimetry, some researchers have made efforts to determine ENDE value mainly at isocenter by different detectors, accelerators, electron doses, energies, etc., as given in the introduction. Since there is no standardized PN and EN dosimetry method yet introduced, verification of the results of such studies requires further efforts which is not in the scope of this paper. However, having two sets of seven ENDE (µSv.Gy^–1^) responses and seven PNDE (µSv.Gy^–1^) recently obtained in the same accelerator at the same exposure conditions provided an excellent opportunity for comparison^4^. Based on the studies made, some general findings include:

1. The ENDE and PNDE studies made so far by different researchers, dosimeters only detects total neutron dose and commonly placed at the isocenter without separating enegy-specific dose components. Accordingly, a general concluding statement can be made that “Most neutron dosimeters used in PNDE studies worldwide, even if they are sensitive to the whole PN or EN energy spectrum, detect total neutrons emitted from the beam and from those scattered back from ground, walls, etc. with no potential to separate thermal, epithermal and fast neutron doses of the beam and of albedo neutrons”. Therefore, the ENDE and PNDE reported so far in the literature even if they have been well studied are over-estimates of the beam neutron doses and should be carefully considered for EN and PN reaction cross section determination, PN- or EN-SPC risk estimation to patients, etc.
2. By using the “miniature dosimeter/spectrometer”, seven ENDE (µSv.Gy^–1^) responses were determined for thermal (beam), albedo thermal, total thermal, total epithermal, total fast, total thermal + epithermal, and total thermal + epithermal + fast ENs. The values for the peak of ENDE (µSv.Gy^–1^) responses at the isocenter are respectively 21 ± 2.63, 43 ± 3.70, 64 ± 6.33, 32 ± 3.90, 112.00, 96 ± 10, and 208 ± 10.23 (µSv.Gy^–1^). Such responses are unique and exclusive to this study and can only be determined by dosimeters capable of separating different energy-specific neutrons like “miniature neutron dosimeter/spectrometer.
3. Having obtained two sets of seven ENDE (µSv.Gy^–1^) per Gray of 20 MeV electron dose and seven PNDE (µSv.Gy^–1^) per Gray of 18 MV X-rays responses obtained at quite similar exposure conditions, the ratios of PNDE (µSv.Gy^–1^)/ENDE (µSv.Gy^–1^) for the seven neutron responses are 28.60 times (beam thermal), 20.93 times (albedo thermal), 23.43 times (total thermal), 32.90 (total epithermal), 19.20 times (total fast), 26.56 (total thermal + epithermal), and 22.60 times (total thermal + epithermal + fast) neutrons. It can be well observed that the ratios of PNDE relative to ENDE are about 20 to 33 times higher since the cross section for ENs are much lower than that of PNs.
4. The Sohrabi “miniature neutron dosimeter/spectrometer” and Stripe PND applied in the two sets of studies seem to have fulfilled the requirements for dosimetry on the whole-body phantom surface and in PE phantom depths and are well separated in terms of energy in seven ENDE (µSv.Gy^–1^) PNDE (µSv.Gy^–1^) responses.
5. While the seven ENDE (µSv.Gy^–1^) responses have unique and exclusive values for patient dosimetry and other purposes, the comparison with seven PNDE (µSv.Gy^–1^) responses has been an added value since it is not possible to obtain similar data in the literature for comparison. While this is not a part of the patient treatment protocol, it proves how patients and others can be protected against the ENDE values obtained.
6. The method also has applications for advanced individual neutron dosimetry of workers, what present methods fail to provide, workplace monitoring, neuron beam characterization studies, and any other applications requiring detailed energy-specific dose determination.
7. One interesting finding is that the EN spectrum and PN spectrum have the same general trends in terms of energy as observed in the studies for ENs^21^ and for PNs^10^.

## Conclusions

Electroneutron (EN) dosimetry/spectrometry was made in the electron beam of 20 MeV Varian Clinac 2100C electron medical accelerator. Sohrabi neutron dosimeters including “miniature neutron dosimeter/spectrometer” and Stripe PNDs were used in obtaining seven major ENDE (µSv.Gy^–1^) responses. This is a breakthrough powerful state-of-the-art neutron dosimetry/spectrometry development in obtaining such detailed energy-specific and tissue-specific ENDE responses as studied for the first time in the world. Comparing the seven ENDE responses of this study with seven PNDE responses of our PNDE studies^4^ showed that the PNDE responses have over 33 times higher values relative to ENDE responses. It was also found that the EN spectrum^21^ and PN spectrum^10^ have the same general energy distribution trends, as discussed in the text above. The methods applied not only is specific to this application but it also has applications in nuclear science and technology in particular in health physics, medical physics, environmental studies, and specifically advanced individual monitoring services.

## Data Availability

All data generated or analysed during this study are included in this published article.
